# TMARg, a Novel Anthraquinone Isolated from *Rubia cordifolia* Nakai, Increases Osteogenesis and Mineralization through BMP2 and β-Catenin Signaling

**DOI:** 10.3390/ijms21155332

**Published:** 2020-07-27

**Authors:** Kyung-Ran Park, Joon Yeop Lee, Bo-Mi Kim, Sang Wook Kang, Hyung-Mun Yun

**Affiliations:** 1 Department of Oral and Maxillofacial Pathology, School of Dentistry, Kyung Hee University, Seoul 02447, Korea; rudfks282@naver.com; 2 National Institute for Korean Medicine Development, Gyeongsan 38540, Korea; chool9090@nikom.or.kr (J.Y.L.); bom0203@nikom.or.kr (B.-M.K.)

**Keywords:** phytomedicine, *Rubia cordifolia* Nakai, TMARg, osteoblast, BMP2, β-catenin

## Abstract

Background: Plant extracts have long been regarded as useful medicines in the treatment of human diseases. *Rubia cordifolia* Nakai has been used as a traditional medicine, as it has pharmacological properties such as antioxidant and anti-inflammatory activity. However, the biological functions of TMARg, isolated from the roots of *R. cordifolia,* in osteoblast differentiation remain unknown. This study was performed to investigate the pharmacological effects and intracellular signaling of TMARg in the osteoblast differentiation of pre-osteoblast MC3T3-E1 cells and mesenchymal precursor C2C12 cells. Methods: Cell viability was evaluated using an MTT assay. Early and late osteoblast differentiation was examined by analyzing the activity of alkaline phosphatase (ALP), and by staining it with Alizarin red S (ARS). Cell migration was determined by using migration assays. Western blot analysis and immunocytochemical analysis were used to examine the intracellular signaling pathways and differentiation proteins. Results: In the present study, TMARg showed no cytotoxicity and increased the osteoblast differentiation in pre-osteoblasts, as assessed from the alkaline phosphate (ALP) staining and activity and ARS staining. TMARg also induced BMP2 expression and increased the p-smad1/5/8-RUNX2 and β-catenin pathways in both MC3T3-E1 and C2C12 cells. Furthermore, TMARg activated mitogen-activated protein kinases (MAPKs) and increased the cell migration rate. In addition, the TMARg-mediated osteoblast differentiation was suppressed by BMP and Wnt inhibitors with the downregulation of BMP2 expression. Conclusion: These findings demonstrate that TMARg exerts pharmacological and biological effects on osteoblast differentiation through the activation of BMP2 and β-catenin signaling pathways, and suggest that TMARg might be a potential phytomedicine for the treatment of bone diseases.

## 1. Introduction

*Rubia cordifolia* Nakai is a flowering plant species from the family of coffee (Rubiaceae), and the roots of the plant have drawn considerable attention as an important medicine due to their potent pharmacological properties, including anti-inflammatory, neuroprotective, anti-oxidant, hepatoprotective, and anti-diabetic properties [1,2,3,4,5]. The roots of the Rubiaceae family plants have also been used as sources of anthraquinones [6]. The derivatives of 1,3,6-trihydroxy-2-methylanthraquinone were only found in some forms of *R. cordifolia* [7]. It was reported that anthraquinone derivatives from *Morinda officinalis* have antiosteoporotic effects in vivo>, and that emodin, an anthraquinone derivative from the roots and bark of the genus Rhamnus, and anthraquinone glycoside aloin induced osteogenic initiation of MC3T3-E1 cells in vitro [8,9,10]. Our group has isolated 1,3,6-trihydroxy-2-methyl-9,10-anthraquinone-3-*O*-(6′-*O*-acetyl)-α-l-hamnopyranosyl-(1→2)-β-d-glucopyranoside (TMARg) from the roots of *R. cordifolia* Nakai. However, its pharmacological effects in osteoblast lineages have not been defined yet.

Major bone diseases, such as osteoporosis and periodontitis, are characterized by abnormalities of bone formation. The defective and excessive bone formation is caused by dysfunctions in proliferation, migration, and differentiation of osteoblast lineages. Osteoblast lineages are specialized cells that were differentiated from mesenchymal stem cells (MSCs) [11]. Osteogenic growth factors, such as BMPs and Wnts, induce osteogenesis in MSCs, leading to bone formation with the synthesis of bone specific proteins and the mineralization of organic bone matrix [11,12,13,14,15]. In pharmacological approaches, such as the use of anabolic agents to stimulate bone formation, parathyroid hormone (PTH) therapy replaces the disadvantages of anti-resorptive agents, such as bisphosphonates and calcitonin [10,16]. However, PTH has limited availability due to being comparatively more expensive and has some other disadvantages associated with it. Therefore, regarding a pharmacological approach using natural compounds, anabolic agents that treat bone diseases, such as osteoporosis, periodontitis, and Paget’s disease, have been identified.

In the present study, we examined the biological mechanisms and intracellular signaling of TMARg on osteogenesis in pre-osteoblast MC3T3-E1 cells and mesenchymal precursor C2C12 cells, using them as in vitro cell systems.

## 2. Results

### 2.1. TMARg Has No Cytotoxicity Effects in Pre-Osteoblasts

**Figure 1:**
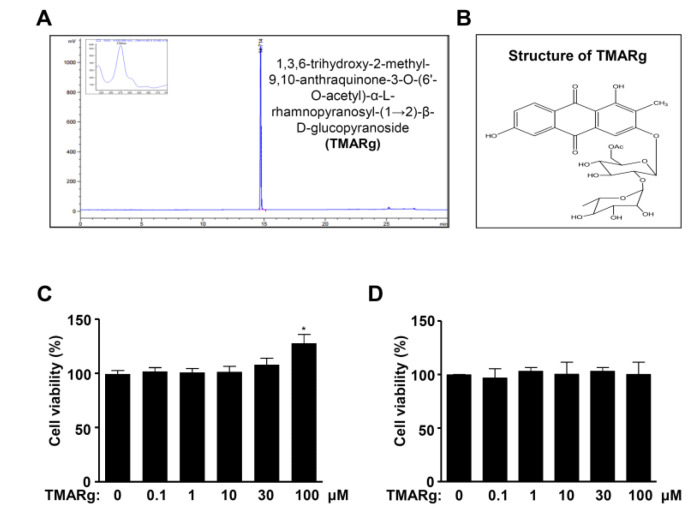
Effects of TMARg regarding cell toxicity in pre-osteoblasts. (**A**) HPLC chromatogram of 1,3,6-trihydroxy-2-methyl-9,10-anthraquinone-3-*O*-(6′-*O*-acetyl)-α-l-rhamnopyranosyl-(1→2)-β-d-glucopyranoside (TMARg) isolated from the roots of *Rubia cordifolia*. (**B**) Chemical structure of TMARg. (**C**,**D**) TMARg was used for the treatment of pre-osteoblasts (C) and C2C12 (D) at concentrations of 0.1, 1, 10, 30, and 100 µM for 24 h. Cell viability was measured by using the MTT assay. The data are representative of the results of three independent experiments. Data represent the means ± SEMs of the experiments. *, *p* < 0.05 was considered significantly different compared to the control.

3,6-Trihydroxy-2-methyl-9,10-anthraquinone-3-*O*-(6′-*O*-acetyl)-α-l-hamnopyranosyl-(1→2)-β-d-glucopyranoside (TMARg) was isolated from the roots of *R. cordifolia* Nakai and the HPLC chromatogram and structure of TMARg are shown in Figure 1A,B. To test the cytotoxicity in pre-osteoblasts, TMARg (0.1, 1, 10, 30, and 100 µM) was used for treatment of pre-osteoblast MC3T3-E1 cells and C2C12 cells for 24 h, and cell viability was investigated using an MTT assay. TMARg did not affect cytotoxicity in the cells (Figure 1C,D). For the next treatment experiments, concentrations of TMARg below 100 μM were used.

### 2.2. TMARg Increases the Staining and Activity of ALP during Osteogenesis of Pre-Osteoblasts

**Figure 2:**
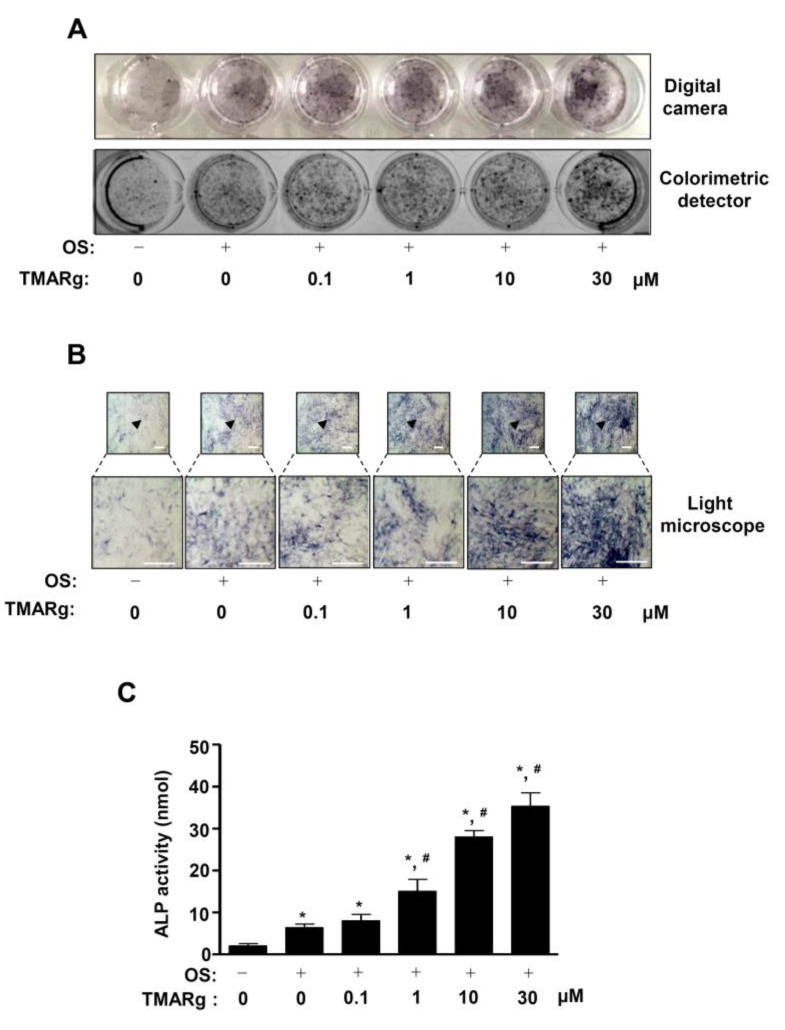
TMARg promotes early osteoblast differentiation. (**A**,**B**) Pre-osteoblasts were seeded onto 24-well plates (2 × 10^4^ cells/well) and were cultured in osteogenic supplement medium (OS) containing 50 µg/mL l-AA and 10 mM β-GP in the absence or presence of TMARg (0, 0.1, 1, 10, and 30 µM) for five days. The levels of ALP staining were detected using a digital camera (upper) and a colorimetric detector (bottom) (A). ALP-stained cells were observed by using a light microscope (bottom: magnified images). The arrowhead indicates the magnified region (B). (**C**) After the cells were seeded onto 48-well plates (1 × 10^4^ cells/well), ALP activity was measured at 405 nm using the Multiskan GO Microplate Spectrophotometer**.** Scale bar: 100 µm. The data are representative of the results of three independent experiments. Data represent the means ± SEMs of the experiments. *, *p* < 0.05 was considered significantly different, compared to the control. #, *p* < 0.05 was considered significantly different, compared to the OS.

To demonstrate the effects of TMARg on osteogenesis, we induced osteoblast differentiation in osteogenic supplement medium (OS) containing 50 µg/mL l-AA and 10 mM β-GP with TMARg. Osteoblast differentiation was observed by staining alkaline phosphatase (ALP); it was used as an early phase marker during osteogenesis, using a digital camera and a colorimetric detector. As shown in Figure 2A, TMARg increased ALP staining in a dose-dependent manner (Figure 2A). ALP-positively-stained cells were also observed using a light microscope (Figure 2B). Under the same conditions, the treatment of TMARg also significantly increased the ALP enzymatic activity in a dose-dependent manner, which was consistent with ALP staining (Figure 2C).

### 2.3. TMARg Increases Mineralized Nodule Formation during Osteogenesis of Pre-Osteoblasts

**Figure 3:**
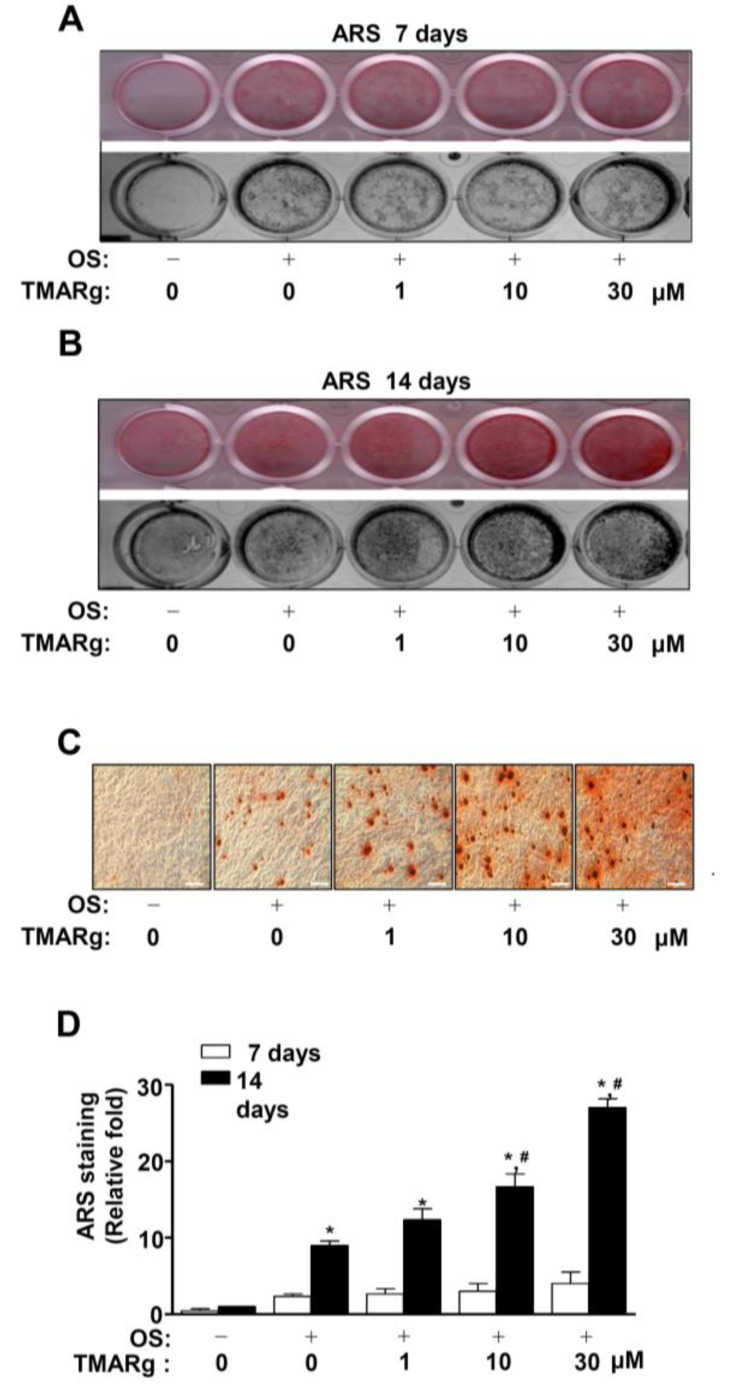
TMARg promotes late osteoblast differentiation. (**A**–**D**) Pre-osteoblasts were seeded onto 24-well plates and were cultured in OS with TMARg (0, 0.1, 1, 10, and 30 µM). Mineralized nodule formation was assessed by using ARS staining at 7 (**A**) and 14 days (**B**). The levels of ARS staining were observed using a scanner (upper) and a colorimetric detector (bottom) (**A**,**B**). Mineralization nodules were observed by using a light microscope (**C**). Stains were eluted with DMSO to quantify the intensity of ARS staining, and this quantification was performed by using the Multiskan GO Microplate Spectrophotometer. The data are represented as relative fold-increases of the control (**C**). The data are representative of the results of three independent experiments. Scale bar: 100 µm. Data represent the means ± SEMs of experiments. *, *p* < 0.05 was considered significantly different, compared to the control. #, *p* < 0.05 was considered significantly different, compared to the OS.

In order to validate the effects of TMARg on osteogenesis, the late osteoblast differentiation was determined by using Alizarin red S (ARS) staining, to assess the degree of matrix mineralization using a scanner and colorimetric detector. A mineralized nodule was visually formed at 14 days, but not at seven days (Figure 3A–C). At 14 days, TMARg increased the matrix mineralization in a dose-dependent manner (Figure 3B,C). To validate the effects of TMARg on late osteoblast differentiation, ARS staining was quantified, and the data significantly revealed the stimulatory effect of TMARg in a dose-dependent manner (Figure 3D).

### 2.4. TMARg Activates BMP2-Smad1/5/8 and β-Catenin Signaling, and Increases RUNX2 Expression during Osteogenesis

**Figure 4:**
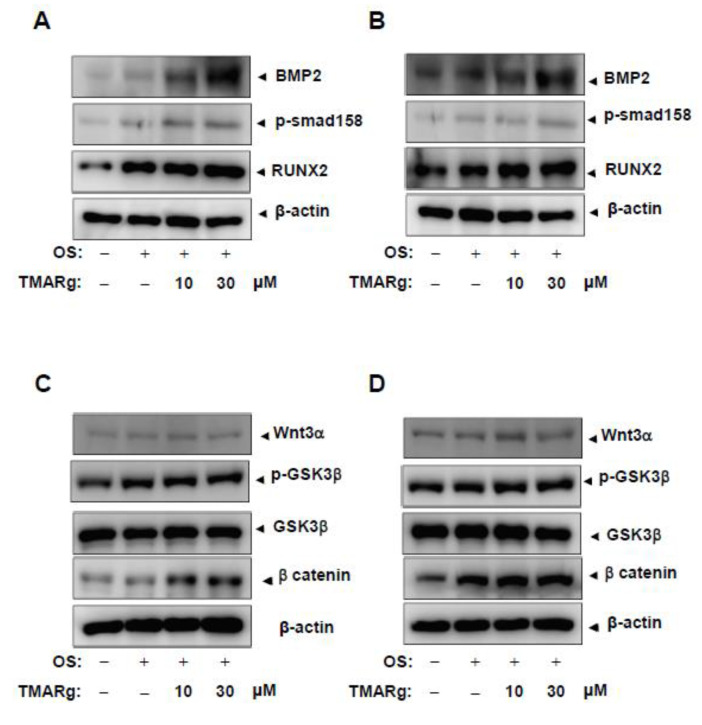
TMARg enhances intracellular BMP2 signaling and β-catenin in osteoblast differentiation. (**A**–**D**) Mesenchymal cells (**A**,**C**) and pre-osteoblasts (**B**,**D**) were cultured in OS with TMARg (10 and 30 µM) for 24 h. BMP2, phospho-Smad1/5/8, RUNX2, and β-actin (**A**,**B**), and Wnt3a, phospho-GSK3 β, GSK3 β, β-catenin, and β-actin (**C**,**D**) were assessed by using Western blot analysis. β-actin was detected on the same sample to normalize the values obtained for the lysates. The data are representative of the results of three independent experiments.

**Figure 5:**
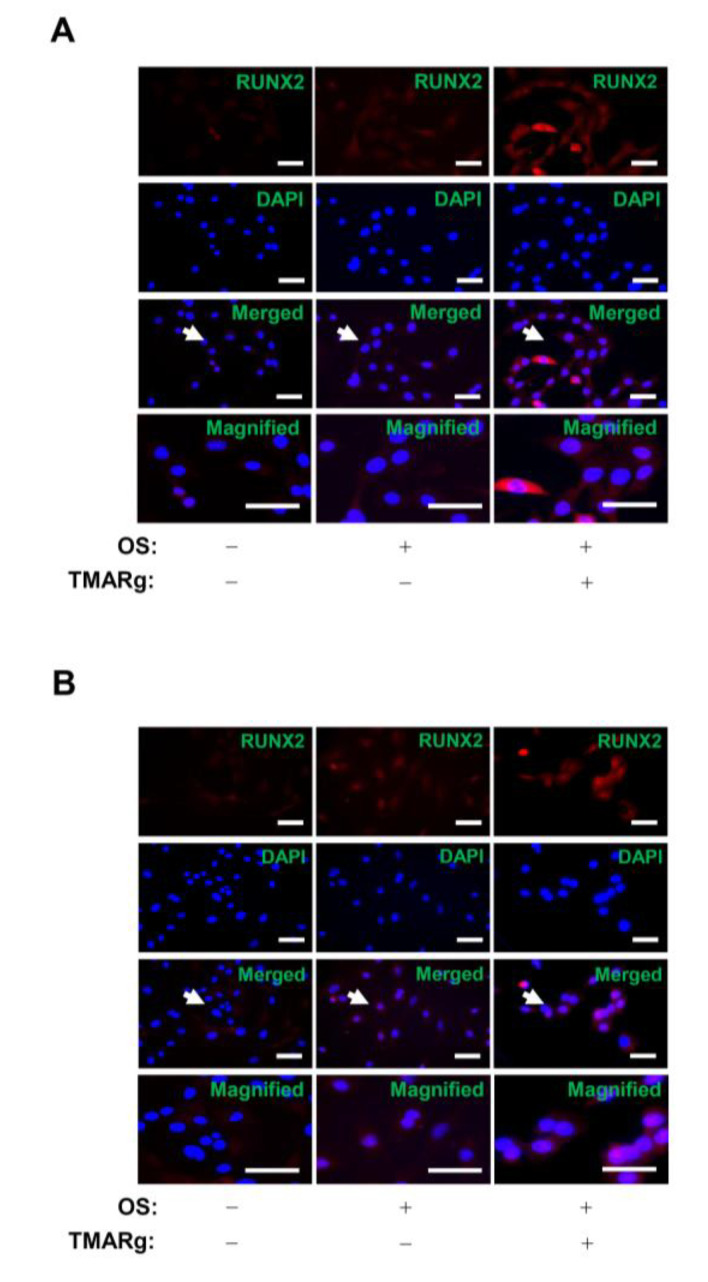
TMARg accumulates the expression of RUNX2 into the nucleus in osteoblast differentiation. (**A**,**B**) After mesenchymal cells (**A**) and pre-osteoblasts (**B**) were cultured in OS with TMARg (30 µM) for 24 h, the cells were fixed and permeabilized. RUNX2 was immunostained with rabbit anti-RUNX2 antibody, followed by Alexa-Fluor 568-conjugated secondary antibody (red). Then, the cells were counterstained with DAPI (blue). The third panel shows the merged images of the first and second panels. The bottom panels show the magnifications of the merged images. The arrow indicates the magnified region. Scale bar: 50 µm. The data are representative of the results of three independent experiments.

To determine the biological mechanism underlying the effect of TMARg on osteogenesis, bone morphogenetic protein (BMP) signaling was examined in mesenchymal precursor and pre-osteoblast cells. TMARg significantly enhanced the expression of BMP2 and increased the phosphorylation of Smad1/5/8 protein and downstream signaling molecules of BMP2, and upregulated the expression of *RUNX2*, a BMP2 target gene, which is a key transcription factor that plays an essential role in osteoblast differentiation (Figure 4A,B). Next, we examined whether TMARg affects β-catenin signaling in osteogenesis. TMARg increased the phosphorylation of GSK3β and the levels of β-catenin in osteoblast differentiation, while TMARg did not affect the expression of Wnt3a. We further confirmed the expression of RUNX2 in the nucleus after the treatment with TMARg in mesenchymal precursor and pre-osteoblast cells, using immunocytochemistry. Immunofluorescence staining revealed that TMARg increased the nuclear expression of RUNX2 in osteoblast differentiation (Figure 5A,B).

### 2.5. TMARg Activates MAPKs and Increases Cell Migration during Osteogenesis

**Figure 6:**
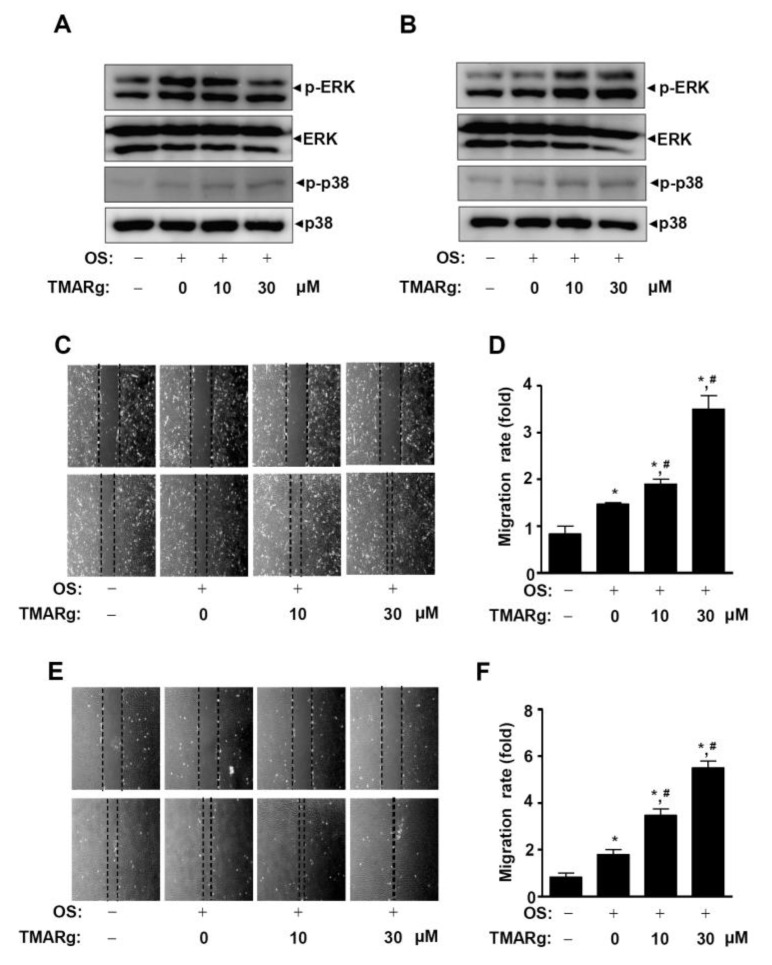
TMARg activates MAPKs signaling and promotes the cell migration rate in osteoblast differentiation. (**A**,**B**) After mesenchymal cells (**A**) and pre-osteoblasts (**B**) were cultured in OS with TMARg (10 and 30 µM) for 24 h, phospho-ERK1/2, ERK1/2, phospho-p38, and p38 were analyzed using Western blot analysis. (**C**–**F**) Cell migration in mesenchymal cells (**C**,**D**) and pre-osteoblasts (**E**,**F**) was observed under a phase contrast microscope (**C**,**E**), and the cell migration rate (fold) was measured by the area enclosing the spreading cell population and expressed as a bar graph normalized to the control (**D**,**F**). The data are representative of the results of three independent experiments. Data represent the means ± SEMs of experiments. *, *p* < 0.05 was considered significantly different, compared to the control. #, *p* < 0.05 was considered significantly different, compared to the OS.

We next examined whether mitogen-activated protein kinases (MAPKs) are involved in the regulation of TMARg-mediated osteoblast differentiation. The results showed that TMARg stimulates the phosphorylation of ERK1/2 in pre-osteoblast cells but not in mesenchymal precursor cells, while TMARg increased the activation of p38 in both pre-osteoblast and mesenchymal precursor cells (Figure 6A,B). To investigate whether TMARg affected the cell migration of pre-osteoblasts due to MAPKs, a wound healing migration assay was carried out. TMARg significantly enhanced the cell migration rate in a dose-dependent manner in mesenchymal precursor cells (Figure 6C,D). Pre-osteoblasts revealed a more evident increase than that of mesenchymal precursor cells (Figure 6E,F).

### 2.6. TMARg Promotes Osteogenesis via the BMP2 and β-Catenin Pathways during Osteogenesis

**Figure 7:**
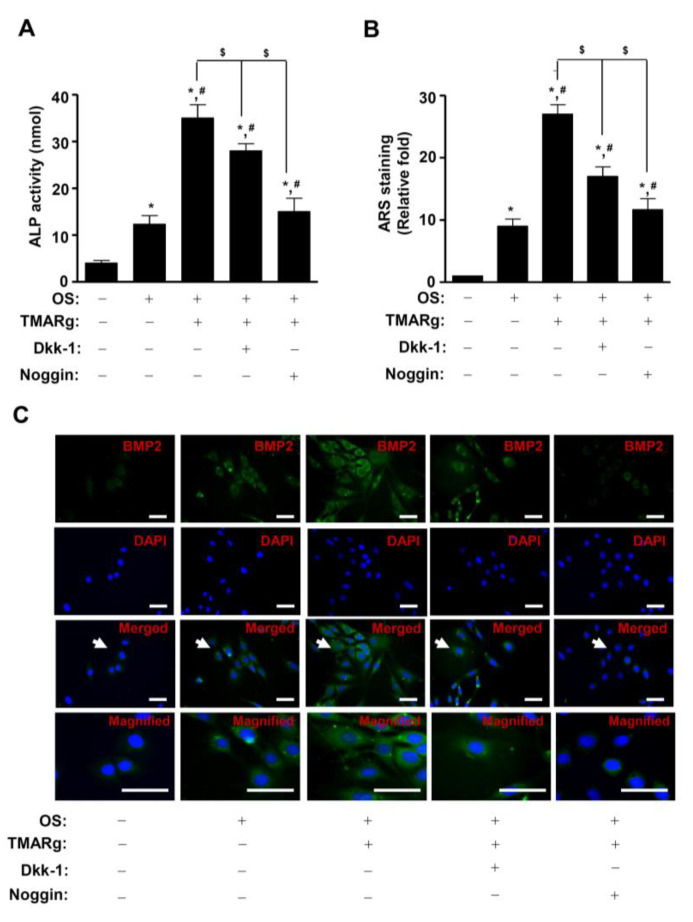
Inhibition of BMP2 and β-catenin signaling attenuates osteoblast differentiation using TMARg treatment. (**A**) The cells were seeded onto 48-well plates and were cultured in OS with TMARg (30 µM) in the absence or presence of Dkk-1 (0.5 µg/mL) and noggin (10 µg/mL) for 5 days. ALP activity was measured by using ALP activity assays. (**B**) The cells were seeded onto 24-well plates and differentiated for 14 days. Mineralized nodule formation was assessed using ARS staining. (**C**) BMP2 was immunostained with rabbit anti-BMP2 antibody, followed by Alexa-Fluor 488-conjugated secondary antibody (green). Then, the cells were counterstained with DAPI (blue). The bottom panels show the magnification of the merged images. The arrow indicates the magnified region. Scale bar: 50 µm. The data are representative of the results of three independent experiments. Data represent the means ± SEMs of experiments. *, *p* < 0.05 was considered significantly different, compared to the control. #, *p* < 0.05 was considered significantly different, compared to the OS. $, *p* < 0.05 was considered significantly different, compared to the OS + TMARg.

To investigate the functional consequence of the TMARg-mediated BMP2 and β-catenin pathways on osteogenesis, TMARg was treated in the presence or absence of BMP2 antagonist, noggin, and Wnt/β-catenin signaling inhibitor Dickkopf-1 (Dkk-1). Results showed that the pretreatment of noggin significantly inhibited TMARg-induced ALP enzymatic activity and mineralized nodule formation, while the pretreatment of Dkk-1 only partially suppressed these (Figure 7A,B). Consistent with the preceding findings, noggin and Dkk-1 also attenuated the TMARg-stimulated BMP2 expression in osteoblast differentiation (Figure 7C).

## 3. Discussion

Many studies have demonstrated that natural compounds increase osteoblast differentiation through signaling pathways, transcription factors, and bone-specific proteins [17,18]. As anabolic agents, natural compounds have been identified to treat bone diseases, such as osteoporosis, periodontitis, and Paget’s disease. [19,20]. In the present study, we first identified the efficacy of TMARg in osteoblast differentiation. The process of osteogenesis from mesenchymal cells and pre-osteoblasts is driven by a complex series of biological processes initiated by the migration of mesenchymal cells to bone formation sites and their subsequent proliferation, and lineage commitment into pre-osteoblasts, leading to the maturation of osteoblasts and the mineralization of the organic bone matrix [21,22]. If this process is abnormal, it leads to bone diseases, such as osteoporosis and periodontal disease [13,14,15]. In the early osteoblast differentiation, the expression of ALP is upregulated, which induces and regulates specific osteoblast genes. In late osteoblast differentiation, the extracellular bone matrix is mineralized by calcium deposition [23,24,25]. The present results revealed that TMARg-mediated osteoblast differentiation upregulated ALP expression and significantly enhanced its enzymatic activity; TMARg also increased mineralized nodule formation. These data suggest that TMARg induces early and late osteoblast differentiation of pre-osteoblasts.

BMP proteins have been known to stimulate osteogenesis and bone formation by inducing osteoblast differentiation [26,27]. BMP2 interacts with BMP receptor IA (BMPRIA) or BMPRIB, and BMPRII. BMP2-mediated BMPR signaling induces the phosphorylation of Smad1/5/8, the interaction between phosphorylated Smad1/5/8 and Smad4, the nuclear translocation of the complexes, and then the regulation of gene expression, such as that of the *RUNX2* gene which is an essential transcription factor for osteoblast differentiation [28,29,30]. In the present study, TMARg increased the expression of BMP2 and the phosphorylation of Smad1/5/8, which was followed by the expression of RUNX2. The involvement of BMP2 in osteoblast differentiation and mineralization was also clarified by its inhibitor, noggin. It was also reported that ALP expression is upregulated by Smad1/5/8 and RUNX2 signaling in osteogenesis [31,32]. These data suggest that TMARg regulates the BMP2/Smad1/5/8 pathway in osteoblast differentiation.

β-catenin signaling is also the main mechanism of osteogenesis and bone formation [33]. In vitro and in vivo studies showed that the activation of the canonical Wnt/β-catenin pathway promotes osteoblast differentiation and mineralization [25,34,35,36]. The canonical Wnt3a binds to Frizzled and LRP5/6 receptors, and induces the stabilization of cytoplasmic β-catenin by inhibiting GSK3β [37]. Consequently, β-catenin is accumulated in the cytoplasm and translocated into the nucleus to regulate gene transcription [38,39]. In the present study, TMARg increased the phosphorylation of GSK3β and the stabilization of β-catenin by inactivating GSK3β. It was reported that β-catenin signaling increases BMP2 expression in mesenchymal cells and pre-osteoblasts, and also that RUNX2 integrates the BMP2 and Wnt/β-catenin signaling pathways during osteogenesis [40,41,42,43]. In addition, our data revealed that Dkk-1 abolished TMARg-mediated osteoblast differentiation and BMP2 expression. These results suggest that TMARg induces the functional cross talk that integrates BMP2 and β-catenin and stimulates osteoblast differentiation and mineralization through the BMP2/Smad1/5/8 and Wnt/β-catenin signaling pathways.

The non-canonical BMP2 signaling pathway also activates MAPKs, such as ERK1/2, p38, and JNK1/2 to regulate osteogenic transcription factors, such as RUNX2 [44,45], and proliferation and migration of mesenchymal cells and pre-osteoblasts [46,47,48,49]. In the present study, TMARg only increased the phosphorylation of p38 in mesenchymal cells, while TMARg increased the phosphorylation of ERK1/2 and p38 in pre-osteoblasts. This might be the difference in ERK1/2 activation in both mesenchymal cells and pre-osteoblasts, since mesenchymal cells are committed to actively proliferate into pre-osteoblasts and further differentiate into non-proliferating osteoblasts, to cause maturation and mineralization [21,22,50]. In the present study, TMARg promoted an increase in the cell migration rate that leads to osteoblast differentiation. Thus, our data suggest that TMARg promotes cell migration through MAPK signaling in mesenchymal cells and pre-osteoblasts.

In conclusion, we originally demonstrated that TMARg isolated from the roots of *R. cordifolia* stimulated osteoblast differentiation with increases in ALP activity, nodule formation, and migration through the BMP2 and β-catenin signaling pathways. Therefore, our findings provide novel evidence on the biological mechanisms of TMARg in osteogenesis that give rise to a great potential regarding the bone patho-physiology. TMARg may also be a phytotherapeutic compound that can be used to treat bone diseases, such as osteoporosis and periodontal disease.

## 4. Materials and Methods 

### 4.1. Plant Material

The stem bark of root of *Rubia cordifolia* Nakai was purchased from commercial herbal market, Daegu, Republic of Korea. The materials were confirmed taxonomically by Chong-Won Kim (College of Pharmacy, Catholic University of Daegu, Republic of Korea). A voucher specimen (CUDP 02001) has been deposited at the College of Pharmacy, Catholic University of Daegu.

### 4.2. Extraction and Isolation of TMARg

The dried roots of *Rubia cordifolia* Nakai (10 kg) were extracted with refluxed 100% MeOH for 8 h (500 mL × 3). The MeOH extract (1048.4 g) was solvent partitioned with distilled water and EtOAc. The EtOAc soluble fraction (180 g) was applied to a silica gel column chromatography and eluted with a gradient CH_2_Cl_2_ and MeOH (25:1 to 6:1) to yield ten fractions (RCE 1–RCE 10). The fraction RCE 7 (1.3 g) was subjected to reversed phase column chromatography with 25% aqueous MeOH to give active compound (301.1 mg). The chemical structure of active compound was identified as an anthraquinone, 1,3,6-trihydroxy-2-methyl-9,10-anthraquinone-3-*O*-(6′-*O*-acetyl)-α-l-hamnopyranosyl-(1→2)-β-d-glucopyranoside (TMARg).

### 4.3. 1,3,6-Trihydroxy-2-Methyl-9,10-Anthraquinone-3-O-(6′-O-Acetyl)-α-l-Hamnopyranosyl-(1→2)-β-d-Glucopyranoside (TMARg)

Yellow powder; ESI-MS *m*/*z* = 621.2 [M + H]^+^, molecular formula C_29_H_32_O_15_; ^1^H-NMR (500 MHz, pyridine-*d*_5_) *δ* 8.37 (1H, d, *J* = 8.4 Hz, H-8), 8.05 (1H, s, H-5), 7.93 (1H, s, H-4), 7.46 (1H, dd, *J* = 2.4, 8.4 Hz, H-7), 6.56 (1H, s, H-1′), 5.86 (1H, d, *J* = 7.6 Hz, H-1″), 2.58 (3H, s, COCH_3_), 2.07 (3H, s, H-11), 1.73 (3H, s, H-6″); ^13^C-NMR (125 MHz, pyridine-*d*_5_) *δ* 187.7 (C-9), 183.2 (C-10), 171.5 (-COCH_3_), 165.6 (C-6), 163.1 (C-1), 161.5 (C-3), 136.9 (C-10a), 133.3 (C-4a), 130.0 (C-8), 126.0 (C-8a), 122.5 (C-7), 122.4 (C-2), 114.3 (C-5), 112.1 (C-9a), 106.7 (C-4), 102.6 (C-1″), 99.6 (C-1′), 79.6 (C-3′), 78.1 (C-2′), 76.1 (C-5′), 74.6 (C-4″), 73.1 (C-2″), 73.0 (C-3″), 71.9 (C-4′), 70.6 (C-5″), 64.8 (C-6′), 21.2 (-COCH_3_), 19.5 (C-6″), 9.9 (C-11).

### 4.4. Nuclear Magnetic Resonance (NMR)

Nuclear magnetic resonance (NMR) experiments were performed on a JEOL ECX-500 spectrometer (^1^H, 500 MHz; ^13^C, 125 MHz; JEOL Ltd., Japan). All chemical shifts were referenced relative to the corresponding signals (δ_H_ 3.31/δ_C_ 49.15 for CD_3_OD). Electron ionization mass spectrometer (EI-MS) data were obtained using micromass spectrum (AUTOSPEC, UK). High performance liquid chromatography (HPLC) was performed using Agilent 1200 series (Agilent Technologies, CA, USA). Open column chromatography (CC) was carried out over a silica gel 60 (70–230 mesh, 230–400 mesh ASTM, Merck, Darmstadt, Germany) and sephadex LH-20 gel (GE Healthcare, Uppsala, Sweden). Pre-coated silica gel 60 F_254_ (Merck) were used for thin-layer chromatography (TLC). 

### 4.5. Culture of Pre-Osteoblast MC3T3E-1 Cells and Mesenchymal Precursor C2C12 Cells, and Osteoblast Differentiation

Pre-osteoblast MC3T3E-1 cells (#CRL-2593) purchased from the American Type Culture Collection (ATCC) (Manassas, VA, USA) were kindly provided by the Bioevaluation Center (Korea Research Institute of Bioscience and Biotechnology, Republic of Korea). The cells were cultured in *α*-minimum essential medium (*α*-MEM) without l-ascorbic acid (WELGEME, Inc., Repubic of Korea) supplemented with 10% fetal bovine serum (FBS), penicillin (100 units/mL), and streptomycin (100 μg/mL) at 37 °C in a humidified atmosphere of 5% CO_2_ and 95% air. Osteoblast differentiation was induced by changing to osteogenic supplement medium (OS) containing 50 µg/mL l-ascorbic acid (l-AA) and 10 mM β-glycerophosphate (β-GP) (Sigma-Aldrich, St. Louis, MO, USA). The medium was replaced every 2 days during the incubation period. Mesenchymal precursor C2C12 cells (#CRL 1722, ATCC, Manassas, VA) kindly provided from Dr. Ki Choon Choi (National Institute of Animal Science, Republic of Korea) were cultured in DMEM (Gibco BRL, Grand Island, NY) supplemented with 10% FBS, penicillin (100 units/mL), and streptomycin (100 μg/mL) at 37 °C in a humidified atmosphere of 5% CO_2_ and 95% air. Osteoblast differentiation was induced by changing to OS containing 50 µg/mL l-AA and 10 mM β-GP (Sigma-Aldrich, St. Louis, MO) with recombinant BMP2 (100 ng/mL) (R&D Systems, Minneapolis, MN). The medium was replaced every 2 days during the incubation period. TMARg was dissolved in 100% DMSO, and the stock solution is diluted at 1:1000. A final concentration of 0.1% DMSO was used as the control.

### 4.6. MTT Assay

Cell viability was measured using a 3-[4,5-dimethylthiazol-2-yl]-2,5-diphenyltetrazolium bromide (MTT) assay to detect NADH-dependent dehydrogenase activity, as previously described [51]. 

### 4.7. Western Blot Analysis 

Western blot analysis was carried out as previously described [52]. Briefly, equal amounts of proteins (20 µg) transferred to a polyvinylidene fluoride (PVDF) membrane (Millipore, Bedford, MA, USA) were blocked for 1 h at room temperature and incubated overnight at 4 °C with the primary antibodies. The membrane was incubated with diluted horseradish peroxidase (HRP)-conjugated secondary antibodies (1:10,000, Jackson ImmunoResearch, West Grove, PA) for 2 h at room temperature was treated with the enhanced chemiluminescence (ECL) kit (Millipore, Bedford, MA). The bound antibodies were detected using an enhanced chemiluminescence (ECL) kit and the ProteinSimple detection system (ProteinSimple Inc., Santa Clara, CA, USA).

### 4.8. Cell Migration Assay

Cell migration was accessed using an in vitro wound healing assay as previously described [53]. Briefly, the cells were wounded with a 200 µL pipette tip and cultured in the absence and presence of TMARg (10, and 30 µM) for 24 h at 37 °C in a humidified atmosphere of 5% CO_2_ and 95% air. Cell migration was observed under light microscopy and cell migration rate was quantified. 

### 4.9. Alkaline Phosphatase (ALP) Staining Assay

Cells were washed with 1× PBS and then fixed in 10% formalin for 15 min at room temperature. After washing with distilled water, the cells were incubated with substrate solution for the reaction of ALP at 37 °C for 1 h, followed according to the manufacturer’s protocol (Takara Bio Inc., Tokyo, Japan). The ALP staining was detected by a digital camera and colorimetric detector (ProteinSimple Inc., Santa Clara, CA, USA).

### 4.10. ALP Activity Assay

ALP activity was performed according to the manufacturer’s protocol using alkaline phosphatase activity colorimetric assay kit (Biovision, Milpitas, CA, USA). The absorbance was measured at 405 nm using the Multiskan GO Microplate Spectrophotometer (Thermo Fisher Scientific, Waltham, MA, USA).

### 4.11. Alizarin Red S (ARS) Staining

Cells were fixed in 10% formalin for 15 min and rinsed with distilled water. Cells were stained with 2% Alizarin red S (pH 4.2) (Sigma-Aldrich) for 20 min with gentle agitation. The level of Alizarin red S staining was observed using a scanner and colorimetric detector (ProteinSimple Inc., Santa Clara, CA, USA). After scanning the stained wells, stains were dissolved in 100% DMSO and the absorbance was measured at 590 nm using the Multiskan GO Microplate Spectrophotometer (Thermo Fisher Scientific).

### 4.12. Immunocytochemistry 

Cells were fixed with 10% formaline for 15 min at room temperature, permeabilized with 0.2% Triton X-100 in 1× PBS for 20 min, and blocked with 3% BSA diluted in PBS for 1 h and incubated with the specific primary antibodies overnight at 4 °C. Subsequently, the cells were incubated with an anti-rabbit secondary antibody labeled with Alexa-Fluor 488 or Alexa-Fluor 568 (1:500 dilution, Invitrogen, Carlsbad, CA) for 2 h at room temperature. Next, the cells were incubated with 4′,6-diamidino-2-phenylindole (DAPI) (Sigma-Aldrich) for 10 min at room temperature. The cells were washed three times, mounted, and viewed on a confocal microscope (K1-Fluo Confocal Laser Scanning Microscope, Daejeon, Republic of Korea).

### 4.13. Statistical Analysis

The data were analyzed using Prism Version 5 program (GraphPad Software, Inc., San Diego, CA, USA). All numeric values are presented as the means ± SEMs. The statistical significance of data was determined using a Student’s unpaired *t*-test. A value of *p* < 0.05 was considered to statistically significant.

## Abbreviations

ALP	Alkaline phosphatase
ARS	Alizarin red S
β-GP	β-glycerophosphate
l-AA	l-ascorbic acid
MAPKs	Mitogen-activated protein kinases
MSCs	Mesenchymal stem cells
MTT	3-[4,5-dimethylthiazol-2-yl]-2,5-diphenyltetrazolium bromide
OS	Osteogenic supplement
RUNX2	Runt-related transcription factor 2
TMARg	1,3,6-trihydroxy-2-methyl-9,10-anthraquinone-3-O-(6′-O-acetyl)-α-L-hamnopyranosyl-(1→2)-β-D-glucopyranoside
